# Domain Swapping between AtACS7 and PpACL1 Results in Chimeric ACS-like Proteins with ACS or C_β_-S Lyase Single Enzymatic Activity

**DOI:** 10.3390/ijms24032956

**Published:** 2023-02-03

**Authors:** Chang Xu, Lifang Sun, Yuanyuan Mei, Gongling Sun, Wenjing Li, Dan Wang, Xin Li, Ning Ning Wang

**Affiliations:** 1 College of Life Sciences, College of Agricultural Sciences, Tianjin Key Laboratory of Protein Sciences, Nankai University, Tianjin 300071, China; 2 State Key Laboratory of Medicinal Chemical Biology, Frontiers Science Center for Cell Responses, College of Life Sciences, Nankai University, Tianjin 300071, China

**Keywords:** ethylene, ACC synthase, C_β_-S lyase, dual-enzymatic activity, AtACS7, PpACL1

## Abstract

The gaseous hormone ethylene plays a pivotal role in plant growth and development. In seed plants, the key rate-limiting enzyme that controls ethylene biosynthesis is ACC synthase (ACS). ACS has, for a long time, been believed to be a single-activity enzyme until we recently discovered that it also possesses C_β_-S lyase (CSL) activity. This discovery raises fundamental questions regarding the biological significance of the dual enzymatic activities of ACS. To address these issues, it is highly necessary to obtain ACS mutants with either ACS or CSL single activity. Here, domain swapping between Arabidopsis AtACS7 and moss CSL PpACL1 were performed. Enzymatic activity assays of the constructed chimeras revealed that, R10, which was produced by replacing AtACS7 box 6 with that of PpACL1, lost ACS but retained CSL activity, whereas R12 generated by box 4 substitution lost CSL and only had ACS activity. The activities of both chimeric proteins were compared with previously obtained single-activity mutants including R6, AtACS7^Q98A^, and AtACS7^D245N^. All the results provided new insights into the key residues required for ACS and CSL activities of AtACS7 and laid an important foundation for further in-depth study of the biological functions of its dual enzymatic activities.

## 1. Introduction

The volatile ethylene is a very ancient phytohormone that exerts profound effects on a wide range of plant growth and developmental processes including seed gemination, root hair formation, fruit ripening, flowering, sex determination, and so on [1,2,3]. It also functions as a stress hormone that plays pivotal roles in plant responses to biotic and abiotic stresses such as pathogen infection, flooding, heat, and salinity [4,5,6,7]. In seed plants, the biosynthesis pathway of ethylene is thoroughly characterized with three catalytic reactions [8]. Among these, the first committed and rate-limiting step that converts *S*-adenosyl-methionine (SAM) to 1-aminocyclopropane-1-carboxylic acid (ACC) is catalyzed by ACC synthase (ACS). The ethylene biosynthesis pathway of seed plants is thus often called the ACC-dependent pathway.

The ACS proteins in seed plants are encoded by a multigene family and belong to the α superfamily of pyridoxal phosphate (PLP)-dependent enzymes that also contain aminotransferases (AATases) and carbon-sulfur lyases [9,10,11]. It is generally believed that all plant *ACS*s originated from *plant-ACS-like* genes that were derived from aspartate aminotransferases (AATase) genes [12,13]. Nevertheless, we found that neither of the two *ACS-like* genes in the genome of early diverging bryophyte *Physcomitrella patens* encode a functional ACS or AATase and one of them (*PpACS-like 1*, *PpACL1*) instead encodes a C_β_-S lyase (CSL) [14]. Particularly, we recently revealed that, in addition to the well-established ACS activity, AtACS7 in Arabidopsis and many ACS proteins in seed plants widely possess CSL activity [15]. Using bioinformatic approaches coupled with biochemical analyses, it was further demonstrated that genuine ACS that can catalyze the conversion from SAM to ACC is uniquely developed in seed plants [15]. These discoveries not only expand our current understanding toward the molecular evolution of *ACS* genes but also open the door to investigating the biological significances of the encoded bifunctional enzyme [16]. To further identify the key domains and residues required for ACS and CSL activities of ACS protein and elucidate the biological functions and relationships of these two activities, it is highly necessary to obtain ACS mutants with either ACS or CSL single enzymatic activity.

It was revealed previously by the sequence alignment of ACS proteins from various plant species, including apple, tomato, and squash, that ACSs share seven highly conserved regions, designated as boxes 1–7 [17]. Each box contains at least eight amino acid residues and shows greater than 95% identity. Among them, box 5 was identified as the active-site center [18]. However, functional annotations of the other boxes remain yet unknown. We performed domain swapping between AtACS7 and PpACL1 and found that the chimeric protein R6, in which the box 2 of AtACS7 was substituted by that of PpACL1, showed CSL but no ACS activity in vitro [15]. Crystal structural comparisons between R6 and AtACS7 pointed to a critical role of the Q98 residue for ACS activity. Consistently, the AtACS7^Q98A^ mutant displayed high CSL activity while retaining its very limited ACS activity [15]. We also found that the AtACS7^D245N^ mutant showed ACS but almost no CSL activity, representing a completely opposite phenotype to R6 and AtACS7^Q98A^ [15].

In this study, we further found that, when box 6 of AtACS7 was replaced with that of PpACL1, the resulting chimeric protein R10 lost ACS activity and only showed CSL activity. By contrast, as shown by the chimeric R12, substituting box 4 of AtACS7 with that of PpACL1 led to a loss of CSL activity but retained ACS activity. The activities of both chimeras were further compared to the previously obtained CSL or ACS single-activity mutants in Xu et al., 2021 [15]. All the results obtained provided new insights into the key domains required for the ACS and CSL activities of AtACS7 and laid an important foundation for further biological characterizations of its dual enzymatic activities.

## 2. Results

### 2.1. Replacing Box 6 of AtACS7 with That of PpACL1 Led to a Loss of ACS Activity but Retains CSL Activity In Vitro

**Figure 1:**
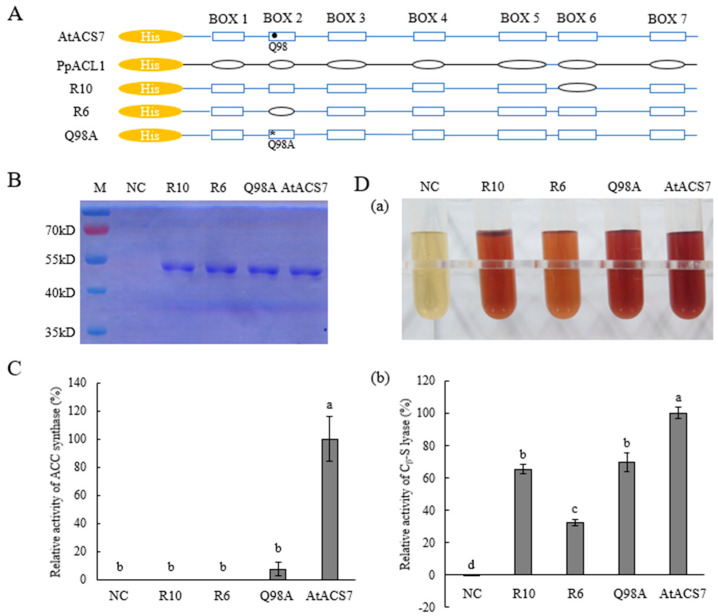
The chimeric protein R10 is a C_β_-S lyase (CSL) single-activity mutant of AtACS7. (**A**) General scheme for the constructions of R10, R6, and AtACS7^Q98A^ with His-tag. The black solid circle indicates position of the wild-type Q98 residue while the asterisk denotes position of its mutated form Q98A. (**B**) Purifications of His-tagged AtACS7, R10, R6, and AtACS7^Q98A^ proteins. (**C**) Measurements of in vitro ACS activities of R10, R6, and AtACS7^Q98A^. (**D**) Determinations of in vitro CSL activities of R10, R6, and AtACS7^Q98A^ by measuring the pyruvate production generated from C_β_-S bond cleavage of L-cystine. Reddish-brown colorations of 2,4-dinitrobenzene-hydrazone generated from pyruvate and 2,4-dinitrobenzene were shown in panel (**a**) while quantitative measurements of CSL activity were displayed in panel (**b**). In both assays, the wild-type AtACS7 protein was used as a positive control and its ACS or CSL activity was normalized to 100%, respectively. Protein extracts from *E. coli* harboring the empty vector served as a negative control (NC). Lower-case letters indicate statistical significance (α = 0.05). One-way ANOVA was followed with Tukey’s test. Data are mean ± standard error (SE) (*n* = 3, biologically independent experiments). Exact *p* values were provided in Supplementary Table S1.

As described in the materials and methods, the chimeric protein R10 was constructed by substituting box 6 of dual-enzymatic AtACS7 with that of the C_β_-S lyase PpACL1 via overlapping PCR (Figure 1A and Figure S1). To investigate whether the domain swapping of box 6 affects the dual enzymatic activities of AtACS7, we first generated a plasmid encoding R10 fused to a His-tag and expressed this plasmid in *E. coli*. The His-R10 recombinant protein was purified using Ni-agarose affinity chromatography and visualized using SDS-PAGE (Figure 1B). The ACS activity of the purified R10 protein that could theoretically convert SAM to ACC was examined using the wild-type AtACS7 as a positive control while the empty vector pET-28a as a negative control (Figure 1C). It was found that, unlike the positive control AtACS7, R10 protein was unable to catalyze the conversion of SAM to ACC (Figure 1C), suggesting that it has no ACS activity.

The in vitro CSL activity assay of R10 was performed to examine whether it could catalyze the cleavage of the C_β_-S bond of substrate L-cystine to produce pyruvate. The generated pyruvate was tested using the 2,4-dinitrobenzene-hydrazine method. The results showed that, as the positive control AtACS7, the purified R10 protein was also able to catalyze the formation of pyruvate which then reacted with 2,4-dinitrobenzene to produce the reddish-brown 2,4-dinitrophenylhydrazone (Figure 1D, panel a). Detailed measurements revealed that R10 retains approximately 70% CSL activity of wild-type AtACS7 (Figure 1D, panel b). Together, these results suggest that the chimeric protein R10 has CSL but not ACS activity in vitro.

When compared to the previously obtained CSL single-activity mutants in Xu et al., 2021 [15], we found that the CSL activity of R10 was remarkably higher than that of R6 but showed no significant difference to that of AtACS7^Q98A^ (Figure 1D). However, while AtACS7^Q98A^ exhibited 6.93% residual ACS activity in vitro, R10 protein has no ACS activity (Figure 1C). Thus, we assume that the chimeric R10 protein is also a suitable CSL single-activity ACS mutant, which can be considered for use in future functional studies.

### 2.2. Substituting Box 4 of AtACS7 with That of PpACL1 Resulted in the Chimeric R12 Protein Having Only ACS Single Activity

**Figure 2:**
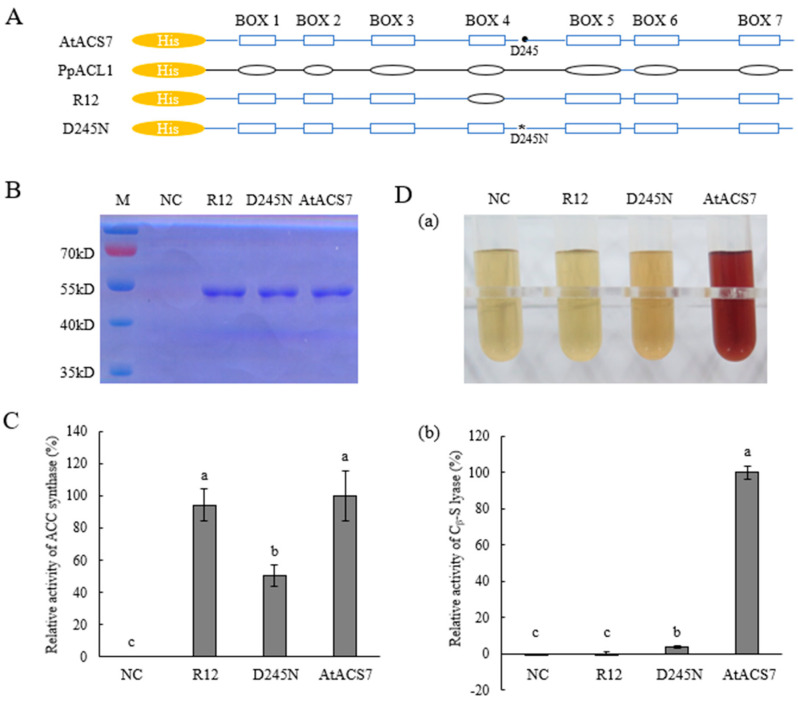
The chimeric protein R12 is an ACS single-activity mutant of AtACS7. (**A**) Schematic diagram illustrating the generation of chimeric protein R12 and the site-directed mutant AtACS7^D245N^ fused with His tag. The black solid circle indicates position of the wild-type D245 residue while the asterisk denotes position of its mutated form D245N. (**B**) SDS-PAGE analysis showing purified proteins of His-tagged AtACS7, R12, and AtACS7^D245N^. (**C**) Determinations of in vitro ACS activities of R12 and AtACS7^D245N^. (**D**) Measurements of the in vitro CSL activities of R12 and AtACS7^D245N^ with L-cystine used as substrate. Reddish-brown colorations of 2,4-dinitrobenzene-hydrazone generated from pyruvate and 2,4-dinitrobenzene were shown in panel (**a**) while quantitative measurements of CSL activity were displayed in panel (**b**). In both assays, the wild-type AtACS7 protein was used as a positive control and its level of activity was normalized to 100%. Protein extracts from *E. coli* harboring the empty vector served as a negative control (NC). Lower-case letters indicate statistical significance (α = 0.05). One-way ANOVA was followed with Tukey’s test. Data are mean ± SE (*n* = 3, biologically independent experiments). Exact *p* values were provided in Supplementary Table S1.

We also generated the chimeric R12 by replacing the conserved box 4 of AtACS7 with that of PpACL1 (Figure 2A and Figure S2) and obtained the purified His-R12 protein using Ni-agarose affinity chromatography (Figure 2B). In vitro dual enzymatic activity assays were performed as mentioned above for His-R10. It was shown that, compared to the wild-type AtACS7, the chimeric protein R12 was able to catalyze the conversion from SAM to ACC (Figure 2C) but failed to convert L-cystine to pyruvate (Figure 2D). These observations indicated that the purified chimeric protein R12 maintained ACS activity but lost CSL activity of the wild-type AtACS7 in vitro.

The dual enzymatic activities of R12 were further compared to ACS7^D245N^, another ACS single-activity mutant of AtACS7 that we obtained previously [15]. The ACS activity of R12 displayed no significant difference with that of the wild-type AtACS7, whereas ACS7^D245N^ only retained approximately half of the ACS activity of AtACS7 (Figure 2C). In addition, although ACS7^D245N^ retained only a residual level of CSL activity, the CSL activity of R12 was even lower (Figure 2D). Together, we infer that R12 is a more ideal candidate of CSL single-activity mutant of AtACS7 that could be used for further functional investigations.

## 3. Discussion

ACS has long been believed to be a single-activity enzyme, catalyzing the rate-limiting step of ethylene biosynthesis from SAM to ACC. However, many growth and developmental defects displayed in higher-order Arabidopsis *acs* mutants were not observed in ethylene-insensitive mutants [19]. The discovery of dual enzymatic activities of ACS prompted questions about the biological significance of its CSL activity as well as the biological relationships between its CSL and ACS activity in plant cells. Additionally, whether the CSL activity of ACS proteins is the causal factor of the complex phenotypes of the *acs* mutants remain yet unknown. Obtaining ACS mutants with either ACS or CSL single activity will greatly help gain clarity on these issues and assist the in-depth study of the biological functions of ACS proteins.

ACS proteins contain seven conserved boxes, among which box 5 was identified as the active-site center while functional analyses of other boxes remain yet unreported [17,18]. In this study, we found that replacing box 6 of AtACS7 with that of PpACL1 led to a loss of ACS activity of AtACS7 and that the resulting chimeric protein R10 has only CSL single activity (Figure 1). By contrast, substituting box 4 of AtACS7 with the corresponding sequence of PpACL1 resulted in the chimeric protein R12 having only ACS single activity (Figure 2). These results imply that box 4 of AtACS7 might be essential for its C_β_-S lyase activity while box 6 seems crucial for maintaining its ACS activity.

Several highly conserved residues have been shown previously to be critical for the catalytic activity of ACS proteins [15,20,21,22], however, none of them are located in the box 4 or box 6 regions. The sequence alignment in this study revealed six mismatches between the box 6 sequences of AtACS7 and R10 (Figure S1). Among them, all the residues of AtACS7 appear to be conserved within genuine ACSs in Arabidopsis except Thr [23]. As collective mutations of these amino acids turned the dual enzymatic AtACS7 to be a CSL single-activity mutant, we infer that these residues, at least the five conserved ones, should be also important for the ACS activity of AtACS7. Of particular note, the loss of ACS activity in R10 might be most likely attributed to the mutation of S313 residue to Met, which seemed to have hindered the substrate binding by occupying a large part of the spaces originally available in the active site (Protein Data Bank code: 7DLW).

Similarly, three mismatches were found between the box 4 sequences of AtACS7 and R12 (Figure S2), among which only the Ser residue was conserved within Arabidopsis genuine ACSs [23]. However, given the fact that domain swapping of this conserved box with that of PpACL1 did not affect the ACS activity of AtACS7 (Figure 2C), we infer that these three residues, including the conserved Ser, may not be as important for ACS activity as previously believed. Instead, they seem to be crucial for the CSL activity of AtACS7. Such replacement with that of PpACL1 might have slightly altered the substrate binding pocket so that it became no longer suitable for L-cystine binding, thus resulting in a complete loss of CSL activity in R12. However, the significance of this hypothesis remains to be established in more detailed studies.

It is also noteworthy that, although replacement of box 2 and box 6 both totally abolished the ACS activity of AtACS7, neither of the resulting chimeras maintained 100% CSL activity of AtACS7. These observations imply that, while residues in these conserved domains play a decisive role in ACS activity, they also affect the level of CSL activity to a certain extent. Compared to R6, R10 has higher CSL activity, suggesting that the substitution in box 6 exerts less effects in CSL activity than that in box 2. Likewise, box 4 seems to play less important roles in maintaining ACS activity than the D245 residue of AtACS7, as the ACS single-activity of R12 was significantly higher than that of ACS7^D245N^. Site-directed mutagenesis in these conserved domains may be carried out in the future to further dissect the most critical residue(s) that is/are required for each enzymatic activity of AtACS7. In addition, analyzing the crystal structures of the mutant proteins and comparing with that of wild-type AtACS7 published earlier by our group will help to further elucidate the specific modes of action of these key residues.

The current study provided valuable gene sources for ACS or CSL single-activity mutants and laid an important foundation for the next study of the biological functions of AtACS7. Pyruvate, the important product of CSL activity of ACS proteins, is the end product of glycolysis and an energy substrate for the Krebs cycle in the mitochondria [24]. It is also indispensable for plants to cope with adverse conditions [25]. In the future, it is necessary to further construct transgenic Arabidopsis overexpressing *R10* or *R12* and verify the single enzyme activity of the encoded AtACS7 mutant protein in the plant cells. An in planta activity assay combined with phenotypic analysis will greatly contribute to a better understanding of the biological functions and relationships of the ACS and CSL activities of ACS proteins.

## 4. Materials and Methods

### 4.1. Construction and Purification of Chimeric Proteins R10 and R12

The chimeras *R10* and *R12* were both generated by overlapping PCR [26] using the coding sequence of *AtACS7* as a template. For the generation of *R10*, two primary PCR products were prepared separately in the first step with *R10-AF* and *R10-AR*, *R10-BF*, and *R10-BR* primers, respectively. In the next step, these intermediate products were mixed, melted, and reannealed at the overlap region that harbors the box 6 sequence of *PpACL1*. The resulting fragment *R10* was amplified in the subsequent PCR cycles by using the outermost primer pair *R10-AF* and *R10-BR*. To obtain the chimeric gene *R12*, two PCR fragments with overlapping ends that contain the box 4 sequence of *PpACL1* were amplified using *R12-AF* and *R12-AR*, *R12-BF*, and *R12-BR* primers, respectively. Subsequently, these products were subjected to overlapping PCR with *R12-AF* and *R12-BR* primers to yield the chimeric fragment *R12*. The primers used are listed in Supplementary Table S2. The resultant fusion products *R10* and *R12* were then cloned into pMD18-T simple vector (Takara, Dalian, China) and transferred into pET28a expression vector via restriction sites *Bam*H I and *Not* I (Takara, Dalian, China). The new expression cassettes were both transformed into *E. coli* BL21 [Rosetta^TM^2 (DE3) plysS] (Biomed, Beijing, China) after being confirmed by sequencing. Purification of the chimeric proteins was carried out using affinity chromatography with His-Trap FF columns (GE Healthcare, Buckinghamshire, UK) according to the manufacturer’s instructions. 

### 4.2. In Vitro ACS Activity Measurement

The in vitro ACS activity was examined using purified proteins of interest with His tag as described previously in Xu et al., 2021 [15]. In brief, 20 μg of the purified protein was added into 460 μL of the ACS assay buffer [50 mM EPPS (pH 8.5), 10 uM PLP, and 2 mM DTT] and 20 ul of 10 mM SAM (Sigma-Aldrich, St. Louis, MO, USA) to a total volume of 500 μL. The mixture was incubated for half an hour at 30 °C. Subsequently, when the reaction was terminated, the ACC formed was converted into ethylene by adding 18 drops of fresh cold mixture of 10% NaClO and saturated NaOH (1:2, *v*/*v*). The vials were then immediately sealed and kept on ice for 5 min. The production of ethylene was measured using a gas chromatograph with a 7890A GC system (Agilent Technologies, Santa Clara, CA, USA). The ACS activity was calculated as the amount of ACC converted from SAM per minute and per microgram protein according to an ACC standard curve.

### 4.3. In Vitro C_β_-S Lyase Activity

The in vitro C_β_-S lyase activity assay was performed using purified proteins of interest with a His tag as described previously in Xu et al., 2021 [15]. Briefly, 100 μg of purified proteins was added to 300 mL of reaction buffer that contained 100 mM PLP, 4 mM L-cystine, and 75 mM potassium phosphate. The mixture was then incubated for 30 min at 30 °C. After the addition of chloroform, the mixture was centrifuged at 12,000 rpm for 10 min at 4 °C. The supernatants were mixed with 2,4-dinitrophenylhydrazine [0.1% (*w*/*v*) in 2 M HCl] and the reaction was terminated by adding 1.5 M NaOH. The contents of pyruvate were quantified by measuring the absorbance at 520 nm and comparing to a standard curve.

### 4.4. Statistical Analysis

All the statistical analyses were performed using one-way ANOVA followed by Tukey’s test with GraphPad prism version 9 (GraphPad Software, La Jolla, CA, USA). Detailed information about statistical analysis values for all the experiments was provided in Supplementary Table S1.

## Data Availability

Not applicable.

## Supplementary Materials

The following supporting information can be downloaded at: https://www.mdpi.com/article/10.3390/ijms24032956/s1.
