# Supplementary material for: Domain Swapping between AtACS7 and PpACL1 Results in Chimeric ACS-like Proteins with ACS or Cβ-S Lyase Single Enzymatic Activity
Source: Int J Mol Sci. 2023 Feb 3;24(3):2956. doi: 10.3390/ijms24032956 (PMC9917878; doi:10.3390/ijms24032956)
Supplement: Supplementary file 1 [file ijms-24-02956-s001.zip › Figures S1 and S2.pdf]

```
AtACS7      MGLPLMMERSSNNNNVELSRVAVSD----THGEDSPYFAGWKAYDENPYDESHNPSGVIQ
PpACL1      --MPMNGDTKDGEFNLKCGRTSLSTRGRRALAPFSPYIKAVNEAKKNPWSL-SNPEGYFV
R10         MGLPLMMERSSNNNNVELSRVAVSD----THGEDSPYFAGWKAYDENPYDESHNPSGVIQ
              :*:  : ...: *:: .*.::*     : .  ***: . :  .:**:.   **.* :

AtACS7      MGLAENQVSFDLLETYLEKKNPEGSMWGSKGAPGFRENALFQDYHGLKTFRQAMASFMEQ
PpACL1      MATAESILSFDLVHDKIRSCREVPA-----------TVGLYGNFRGGERLRNAISRMMER
R10         MGLAENQVSFDLLETYLEKKNPEGSMWGSKGAPGFRENALFQDYHGLKTFRQAMASFMEQ
            *. **. :****:.  :.. .   :              .*: :::* : :*:*:: :**:

AtACS7      IRGGKARFDPDRIVLTAGATAANELLTFILADPNDALLVPTPYYPGFDRDLRWRTGVKIV
PpACL1      TFMG-VEVDPSHICISSGVTAVLDLFFFATCNPGDGCLIPAPYFPAFDNDMSIRNEVIPI
R10         IRGGKARFDPDRIVLTAGATAANELLTFILADPNDALLVPTPYYPGFDRDLRWRTGVKIV
               * ...**.:* :::*.**. :*: *  .:*.*. *:*:**:*.**.*:  *. *  :

AtACS7      PIHCDSSNHFQITPEALESAYQTARDANIRVRGVLITNPSNPLGATVQKKVLEDLLDFCV
PpACL1      PVQPTDTRTYIPTVHEMEEAVLAAESKGIRARMLLVTNPGNPLGTLYPEATLKELLLWAV
R10         PIHCDSSNHFQITPEALESAYQTARDANIRVRGVLITNPSNPLGATVQKKVLEDLLDFCV
            *::  .:. :  * . :*.*  :*.. .**.* :*:***.****:   : .*::** :.*

AtACS7      RKNIHLVSDEIYSGSVFHASE--FTSVAEIVE------NIDDVSVKERVHIVYSLSKDLG
PpACL1      KRGLHVLSDEIYANSKFGPSVDEFVSMEKVTKNAVMEGLLSAETAAELVHTAYGMSKDFG
R10         RKNIHLVSDEIYSGSVFHASE--FTSVAEIVE------NIDDVSVKERVHIVYSLSKDLG
            ::.:*::*****:.* *  *   *.*: ::.:        :.  :. * ** .*.:***:*

                                    BOX6
AtACS7      LPGFRVGTIYSYNDNVVRTARRMSSFTLVSSQTQHMLASMLSDEEFTEKYIRINRERLRR
PpACL1      MNGFRVGCLHTKNKDLLEFWQNMGMFAAVSNDTQHALAIMLEDENFVDKYVKENNRRLKK
R10         LPGFRVGTIYSYNDNVVRTARRMGMFAAVSNDTQHMLASMLSDEEFTEKYIRINRERLRR
            : ***** ::: *.:::.  :.*. *: **.:*** ** **.**:*.:**:: *..**::

AtACS7      RYDTIVEGLKKAGIECLKGNAGLFCWMNLGFLLEKKTKDGELQLWDVILKELNLNISPGS
PpACL1      SYELLTKSFEAANLRYMPACAAMFCWLDLKSLLTEPTFTAEDNLWKEILDECRIVLTPGQ
R10         RYDTIVEGLKKAGIECLKGNAGLFCWMNLGFLLEKKTKDGELQLWDVILKELNLNISPGS
             *: :.:.:: *.:. : . *.:***::*  ** : *  .* :**. **.* .: ::**.

AtACS7      SCHCSEVGWFRVCFANMSENTLEIALKRIHEFMDRRRRF-----------------
PpACL1      ACHYAEPGFFRVCYASMAPASLEIACARLTGFAEKKRRKRKSRDGQLDIDTLRTNH
R10         SCHCSEVGWFRVCFANMSENTLEIALKRIHEFMDRRRRF-----------------
            :** :* *:****:*.*:  :****  *:  * :::**
```

Figure S1: Sequence alignment of AtACS7, PpACL1 and the chimeric protein R10.

```
AtACS7      MGLPLMMERSSNNNNVELSRVAVSD----THGEDSPYFAGWKAYDENPYDESHNPSGVIQ
PpACL1      --MPMNGDTKDGEFNLKCGRTSLSTRGRRALAPFSPYIKAVNEAKKNPWSL-SNPEGYFV
R12         MGLPLMMERSSNNNNVELSRVAVSD----THGEDSPYFAGWKAYDENPYDESHNPSGVIQ
              :*:  : ...: *:: .*.::*     : .  ***: . :  .:**:.   **.* :

AtACS7      MGLAENQVSFDLLETYLEKKNPEGSMWGSKGAPGFRENALFQDYHGLKTFRQAMASFMEQ
PpACL1      MATAESILSFDLVHDKIRSCREVPA-----------TVGLYGNFRGGERLRNAISRMMER
R12         MGLAENQVSFDLLETYLEKKNPEGSMWGSKGAPGFRENALFQDYHGLKTFRQAMASFMEQ
            *. **. :****:.  :.. .   :             .*: :::* : :*:*:: :**:

AtACS7      IRGGKARFDPDRIVLTAGATAANELLTFILADPNDALLVPTPYYPGFDRDLRWRTGVKIV
PpACL1      TFMG-VEVDPSHICISSGVTAVLDLFFFATCNPGDGCLIPAPYFPAFDNDMSIRNEVIPI
R12         IRGGKARFDPDRIVLTAGATAANELLTFILADPNDALLVPTPYYPGFDRDLRWRTGVKIV
               *  ...**.:* :::*.**. :*: *  .:*.*. *:*:**:*.**.*:  *. *  :

                                                     BOX4
AtACS7      PIHCDSSNHFQITPEALESAYQTARDANIRVRGVLITNPSNPLGATVQKKVLEDLLDFCV
PpACL1      PVQPTDTRTYIPTVHEMEEAVLAAESKGIRARMLLVTNPGNPLGTLYPEATLKELLLWAV
R12         PIHCDSSNHFQITPEALESAYQTARDANIRVRGVLITNPGNPLGTLVQKKVLEDLLDFCV
            *::  .:. :  * . :*.*  :*..  .**.* :*:***.****:   :  .*::** :.*

AtACS7      RKNIHLVSDEIYSGSVFHASE--FTSVAEIVE------NIDDVSVKERVHIVYSLSKDLG
PpACL1      KRGLHVLSDEIYANSKFGPSVDEFVSMEKVTKNAVMEGLLSAETAAELVHTAYGMSKDFG
R12         RKNIHLVSDEIYSGSVFHASE--FTSVAEIVE------NIDDVSVKERVHIVYSLSKDLG
            ::.:*::*****:.* *  *   *.*: ::.:       :.  :. * ** .*.:***:*

AtACS7      LPGFRVGTIYSYNDNVVRTARRMSSFTLVSSQTQHMLASMLSDEEFTEKYIRINRERLRR
PpACL1      MNGFRVGCLHTKNKDLLEFWQNMGMFAAVSNDTQHALAIMLEDENFVDKYVKENNRRLKK
R12         LPGFRVGTIYSYNDNVVRTARRMSSFTLVSSQTQHMLASMLSDEEFTEKYIRINRERLRR
            : *****  ::: *.:::.  :.*. *: **.:*** ** **.**:*.:**:: *..**::

AtACS7      RYDTIVEGLKKAGIECLKGNAGLFCWMNLGFLLEKKTKDGELQLWDVILKELNLNISPGS
PpACL1      SYELLTKSFEAANLRYMPACAAMFCWLDLKSLLTEPTFTAEDNLWKEILDECRIVLTPGQ
R12         RYDTIVEGLKKAGIECLKGNAGLFCWMNLGFLLEKKTKDGELQLWDVILKELNLNISPGS
             *: :.:.:: *.:. : . *.:***::*  ** : *  .* :**. **.* .: ::**.

AtACS7      SCHCSEVGWFRVCFANMSENTLEIALKRIHEFMDRRRRF----------------
PpACL1      ACHYAEPGFFRVCYASMAPASLEIACARLTGFAEKKRRKRKSRDGQLDIDTLRTNH
R12         SCHCSEVGWFRVCFANMSENTLEIALKRIHEFMDRRRRF----------------
            :** :* *:****:*.*:  :****  *:  * :::**
```

Figure S2: Sequence alignment of AtACS7, PpACL1 and the chimeric protein R12.
