# Supplementary material for: Domain Swapping between AtACS7 and PpACL1 Results in Chimeric ACS-like Proteins with ACS or Cβ-S Lyase Single Enzymatic Activity
Source: Int J Mol Sci. 2023 Feb 3;24(3):2956. doi: 10.3390/ijms24032956 (PMC9917878; doi:10.3390/ijms24032956)
Supplement: Supplementary file 1 [file ijms-24-02956-s001.zip › Table S2.pdf]

Table S2. Sequences of the primers used in this study.

Name	Primer sequence (5′-3′)
R10-AF	GGATCCATGGGTCTTCCTCTAATGATGGAGAGA
R10-AR	CTGAGTGTCGTTTGAAACTGCTGCAAACATGCCCATCCTTCTCGCTGTCCGA
R10-BF	ATGGGCATGTTTGCAGCAGTTTCAAACGACACTCAGCATATGCTGGCTTCTA
R10-BR	GCGGCCGCTCAAAACCTCCTTCGT
R12-AF	GGATCCATGGGTCTTCCTCTAATGATGGAGAGATCATCAAACAACAACA
R12-AR	AAGAGTGCCCAATGGGTTCCCAGGATTAGTTATGAGCACTCCTCGGAC
R12-BF	ACTAATCCTGGGAACCCATTGGGCACTCTTGTCCAAAAGAAGGTTCTAG
R12-BR	GCGGCCGCTCAAAACCTCCTTCGTCGGTCCATG
